# Five-year Audit of Infectious Diseases at a Tertiary Care Hospital in Karachi, Pakistan

**DOI:** 10.7759/cureus.3551

**Published:** 2018-11-05

**Authors:** Naseem Salahuddin, Muhammad Khalid, Naila Baig-Ansari, Sundus Iftikhar

**Affiliations:** 1 Infectious Diseases, The Indus Hospital, Karachi, PAK; 2 Epidemiology and Public Health, The Indus Hospital, Karachi, PAK; 3 Miscellaneous, The Indus Hospital, Karachi, PAK

**Keywords:** infectious diseases audit pakistan, infectious diseases audit pakistan, infectious diseases prevalence pakistan, infectious diseases burden pakistan

## Abstract

Purpose

To estimate the burden of infectious diseases and the seasonality of mosquito-borne diseases seen at The Indus Hospital, Karachi (TIH).

Methodology

We performed a retrospective data analysis of all infectious diseases (ID) cases, retrieved from medical records over a five-year period starting from 1 January 2012 till 31 December 2016 at The Indus Hospital (TIH), which is a 150-bed, charity-based, tertiary-care health facility. The collected data has been categorized into three groups: (A) public health-related diseases, including community and environmental IDs, i.e., mosquito-borne diseases such as malaria and dengue, respiratory tract infections, diarrheal diseases, typhoid, and hepatitis; (B) systemic infection related IDs that target individual anatomical or physiological systems such as the respiratory tract, urinary tract, skin and soft tissue, and the cardiac system, and lastly, those IDs which are (C) programmatically managed at TIH, namely cases from the tuberculosis (TB), human immunodeficiency virus (HIV)/acquired immunodeficiency syndrome (AIDS), and malaria clinics, and the rabies prevention center. As the study is an audit, ethical approval was waived by the institutional review board (IRB).

Result

Overall data from 71,815 patients were assessed. In the public health group (A), the main bulk of diseases were due to malaria, tuberculosis, respiratory tract infections (upper and lower), and diarrheal diseases in both males and females in descending order; there was preponderance of malaria, respiratory tract infections, and diarrheal diseases in males, and of tuberculosis among females. Among the systemic diseases group (B), urinary tract infections (UTIs) had a disproportionately high incidence, followed by skin and soft tissue infections, while bone and joint infections and diabetic foot had equal incidence. In the programmatic group (C), the highest number of cases seen was dog bites followed by drug-sensitive TB. Overall, the six most common infections were malaria, cases of dog bites, tuberculosis, respiratory tract infections, diarrheal diseases, and hepatitis C. More women than men had TB; diarrheal disease and respiratory tract infections were more common in children. UTIs were the most common systemic infections among both men and women.

Conclusion

There is a great need to have an effective surveillance mechanism of preventable diseases at the national level. Our study highlights the diversity of cases that should direct medical curriculum development, post-graduate training, and health services improvement.

## Introduction

Infectious diseases (IDs) are a distinct group of illnesses that are caused by microorganisms. Their burden is highest amongst the developing world and particularly in Southeast Asia by virtue of the distinctive geography, climate, vectors, population, and country-specific health economics of the region [1]. Tuberculosis (TB), human immunodeficiency virus (HIV), and malaria contribute sizeably to the understanding of well-known ID afflictions; however, the epidemiology, distribution, and incidence of individual IDs in various regions are less well known globally [2].

In the past several decades, new and emerging infections have drawn global attention. The appearance of HIV/AIDS in the 1980s shocked the world as it brought into focus a fast-spreading and ultimately fatal disease, and along with it, previously little-heard-of opportunistic infections surfaced, the most devastating of which was tuberculosis. In addition, emerging and re-emerging mosquito-borne infections such as dengue, chikungunya, zika, and more virulent forms of malaria; ebola in West Africa [3], plague in Madagascar [4], and new and more severe forms of viral respiratory infections have evolved. The most challenging problem confronting physicians today is the treatment of previously easy-to-treat IDs; the emergence of antimicrobial resistance is rendering bacterial infections untreatable [5].

Pakistan faces an enormous burden from IDs, and the trend is on the rise. This is compounded by multiple social and economic factors like poverty, the lack of basic health education and a developed healthcare system, over-population, natural disasters, the internal and external migration of displaced persons, and a lack of effective preventive strategies. Diarrheal and respiratory infections, hepatitis A, B, and C, measles, typhoid fever, and TB are common, while mosquito-borne diseases such as malaria, dengue fever, and chikungunya have been rampant. Rabies following dog bites occurs frequently, though it is underreported. Systemic infections related to particular anatomic and physiologic systems have also contributed to the high burden of disease. Most of the diseases are managed by general physicians or by various specialists, mostly in the private sector.

Baseline comprehensive estimates of the burden of IDs are needed for effective planning and the prioritizing of limited public health resources in each country. Over the last three decades, efforts have been made globally to derive and apply methods to estimate disease burden at population scales. In particular, the Global Burden of Disease (GBD) project has made important progress in this area methodologically and in terms of output estimates, and is based on available evidence that supports healthcare policy-making; however, Pakistan does not have an effective ID surveillance system and, unfortunately, it is off track in meeting the Millennium Development Goals in healthcare [6-7].

Even though incidence rates in Pakistan may vary from city to city, little is known about the types of infection and their burden. This paper seeks to address this gap in our knowledge through an investigation of the data available from the electronic medical records of The Indus Hospital (TIH), which runs an active department of infectious diseases.

Objective

To estimate the disease burden, gender, age frequencies for common IDs, and the seasonality of mosquito-borne diseases seen at TIH.

## Materials and methods

Site of audit

TIH is a 150-bed, private, tertiary-care health facility providing high-quality care free-of-cost. It is located in Korangi town, to the east of Karachi port, and in one of Pakistan’s largest industrial zones. The hospital’s direct catchment population is a multi-ethnic community of approximately 2.5 million people, comprising regional and sub-national migrant settlements adjacent to historical fishing villages along the south-eastern Karachi coast. In 2010-2011, there were over 400 emergency room visits daily, and 300 specialty clinic visits each day. These numbers are fast increasing each year. The ID service runs specialty clinics three times a week, manages admissions, offers consultations in the wards, and also conducts antibiotic stewardship rounds in order to curtail inappropriate antimicrobial prescriptions by other clinical disciplines. At TIH, there are also specialized clinics for infections, where management is performed strictly under WHO guidelines. There are five clinics that perform under programmatic management: for drug-sensitive tuberculosis (DSTB), drug-resistant tuberculosis (DRTB), HIV, malaria, and a rabies prevention center (RPC) from animal bites. The ID service also oversees the abovementioned programmatically run services.

We performed a retrospective data analysis of all cases retrieved from medical records over a five-year period starting from 1 January 2012 till 31 December 2016. Data was documented from electronic case records and laboratory reports available through the locally developed hospital management information system (HMIS). The date of the diagnosis and basic demographic features (age and gender) were entered using MS Excel. The data was then filtered and repetitions were removed. For the purpose of selection, data collection, and analysis, the IDs were categorized into three groups:

(A) Public health-related: Includes commonly seen community and environmental IDs, i.e., mosquito-borne diseases like malaria and dengue, respiratory tract infections, diarrheal diseases, typhoid, and hepatitis. Cases of chikungunya started appearing in 2016, and hence do not appear in our audit. Although TB is considered a public health disease, as it is being managed programmatically at TIH, we have included TB in the tables represented both in public health, as well as programmatic diseases.

(B) Systemic infections indicate IDs related to individual anatomical or physiological systems i.e., lungs, kidneys, bones and joints, soft tissue, liver, and heart. Since TIH does not have a neurology service, neurological infections were not in sufficient numbers to be included in the audit.

(C) Programmatically managed: Indus Hospital conducts a program of drug-sensitive (DS) TB, and drug-resistant (DR) TB since 2008, in a purpose-built TB clinic; the HIV/AIDS clinic functions through support from the national AIDS control program since 2009; the malaria program started in 2012. The TB, AIDS, and malaria programs are all supported through a global fund, while the rabies prevention centre functions through the emergency department of TIH. Recently, a funded program for hepatitis C was also added. These programs manage data in their respective clinics, separately from the HMIS.

Statistical methods

Data were entered and analyzed using SPSS version 21.0 (IBM, Armonk, New York, United States). The distribution of public health, systemic, and programmatic diseases were presented as frequencies, along with percentages for pediatric and adult population entered separately. Furthermore, the gender-wise burden of diseases in both the pediatric and adult populations were shown as frequency and percentage.

Ethical approval

As the study is an audit, ethical approval was waived by the institutional review board (IRB).

## Results

Overall data of 71,815 patients were assessed (Table 1).

**Table 1:** Overall Infectious Disease Frequencies (Excluding Systemic Infections) TB - Tuberculosis HBV - Hepatitis B Virus HCV - Hepatitis C Virus HIV: Human Immunodeficiency Virus

Diseases	Frequency
Malaria	22396
Animal bite	15128
TB	12,352
Respiratory tract infection	10011
Diarrheal diseases	3891
HCV	1817
Dengue	788
HIV	421
Typhoid	257
HBV	186
Total	67,427

**Figure 1:**
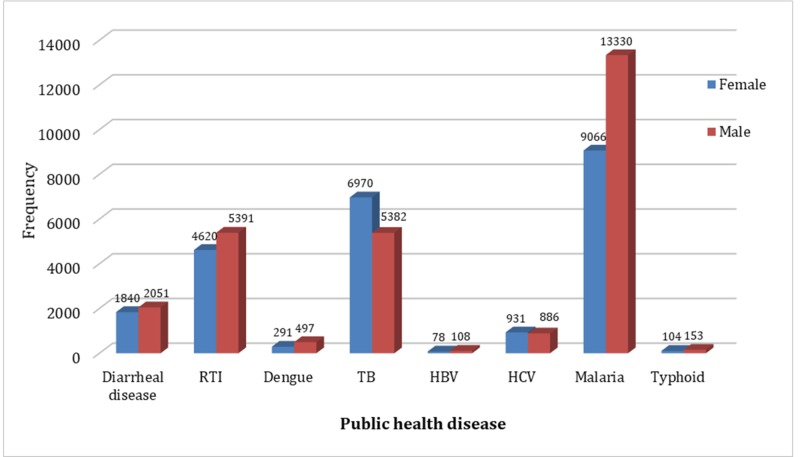
Public Health Diseases RTI - Respiratory Tract Infections TB - Tuberculosis HBV - Hepatitis B Virus HCV - Hepatitis C Virus

The bulk of diseases at this site is due to malaria, tuberculosis, respiratory tract infection (upper and lower, and diarrheal diseases in both males and females. There is preponderance of malaria, respiratory tract infections, and diarrheal diseases in males. Moreover, there is predominance of tuberculosis among females. (Table 2), (Figure 1).

**Table 2:** Commonality of IDs Among All Ages and Both Sexes

Disease	Pediatrics			Adults		
	Female	Male	Total	Female	Male	Total
	n (%)	n (%)	n (%)	n (%)	n (%)	n (%)
Public Health						
Diarrheal diseases	1394 (19.5)	1746 (18.2)	3140 (18.8)	446 (4.6)	305 (2.4)	751 (3.3)
Respiratory tract infection (RTI)	2192 (30.7)	3063 (31.9)	5255 (31.4)	2428 (24.8)	2328 (18.2)	4756 (21.0)
Dengue	111 (1.6)	284 (3.0)	395 (2.4)	180 (1.9)	213 (1.7)	393 (1.7)
Hepatitis B (HBV)	2 (0.0)	4 (0.0)	6 (0.0)	76 (0.8)	104 (0.9)	180 (0.8)
Hepatitis C (HCV)	30 (0.4)	29 (0.3)	59 (0.4)	901 (9.2)	857 (6.7)	1758 (7.8)
Malaria	3313 (46.4)	4342 (45.2)	7655 (45.7)	5753 (58.8)	8988 (70.2)	14741 (65.2)
Typhoid	93 (1.3)	133 (1.4)	226 (1.4)	11 (0.2)	20 (0.2)	31 (0.1)
Total	7135 (100)	9601 (100)	16736 (100)	9795 (100)	12815 (100)	22610 (100)
Systemic Disease						
Bone joint	49 (11.2)	84 (17.8)	133 (14.6)	34 (2.0)	94 (4.8)	128 (3.5)
Soft tissue infection	50 (11.4)	105 (22.2)	155 (17.1)	113 (6.6)	248 (12.8)	361 (9.9)
Diabetic foot	-	-	-	45 (2.6)	148 (7.6)	193 (5.3)
Infective endocarditis	2 (0.5)	6 (1.3)	8 (0.9)	11 (0.6)	15 (0.8)	26 (0.7)
Liver abscess	7 (1.6)	9 (1.9)	16 (1.8)	10 (0.6)	41 (2.1)	51 (1.4)
Tetanus	1 (0.2)	4 (0.8)	5 (0.6)	1 (0.1)	13 (0.7)	14 (0.4)
Urinary tract infections (UTI)	328 (75.1)	264 (55.9)	592 (65.1)	1501 (87.5)	1385 (71.2)	2886 (78.9)
Total	437 (100)	472 (100)	909 (100)	1715 (100)	1944 (100)	3659 (100)
Programmatic Disease						
Animal bite	1054 (32.1)	6235 (84.9)	7289 (43.6)	994 (17.2)	6845 (59.6)	7839 (34.7)
Drug-resistant tuberculosis (DRTB)	99 (3.0)	42 (0.6)	141 (0.9)	310 (5.4)	355 (3.1)	665 (2.9)
Drug-sensitive tuberculosis (DSTB)	2128 (64.9)	1065 (14.5)	3193 (19.1)	4433 (76.6)	3920 (34.1)	8353 (36.9)
Human immunodeficiency virus (HIV)	0 (0.0)	3 (0.0)	3 (0.0)	53 (1)	364 (3.2)	417 (1.8)
Total	3281 (100)	7345 (100)	10626 (100)	5790 (100)	11484 (100)	17274 (100)

**Figure 2:**
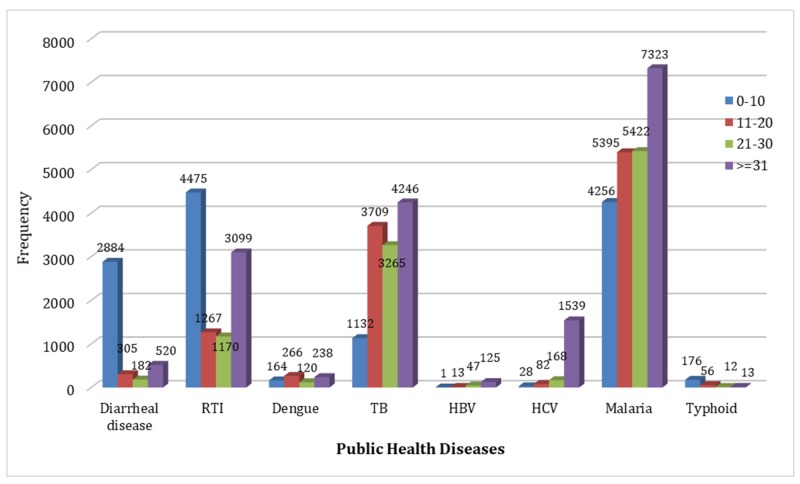
Public Health Diseases RTI - Respiratory Tract Infections TB - Tuberculosis HBV - Hepatitis B Virus HCV - Hepatitis C Virus

Among the 0-10 years age group, respiratory tract infections, malaria, diarrheal diseases, and tuberculosis are most common in descending order of incidence. In the 11-20 years age bracket (adolescence) malaria and tuberculosis have the highest incidence, followed by respiratory tract infections and diarrheal diseases. However, there has been a significant overall decline in these infections among the younger group. Malaria and tuberculosis had the highest incidence among adults aged 21-30 years, followed by respiratory tract infections and a less number of cases of diarrheal diseases. The same infections continue in adults over 30 years; however, there is a sharp rise in the incidence of hepatitis B and C after the third decade. Hepatitis B is now of very low frequency in the general population. Typhoid fever occurs at all ages, but most commonly in young children as reflected in the graph. Even though the Anopheles mosquito is the common vector for both dengue and malaria, the latter by far exceeds dengue among all age groups (Figure 2).

**Figure 3:**
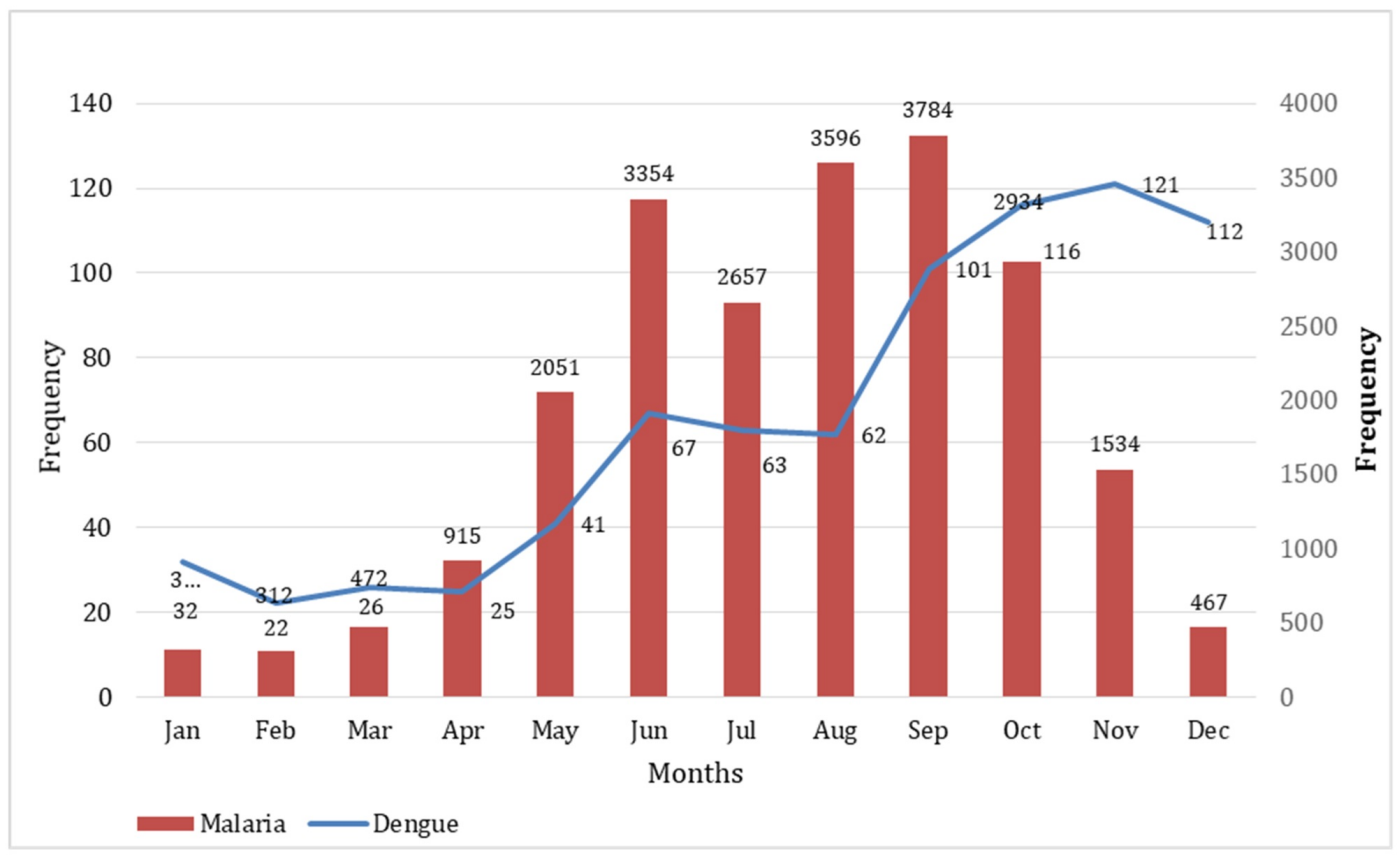
Seasonality of Malaria and Dengue

Although both malaria and dengue occur year round, cases of malaria peak significantly from May to October, while dengue cases occur more in the cooler months and decline after December (Figure 3).

**Figure 4:**
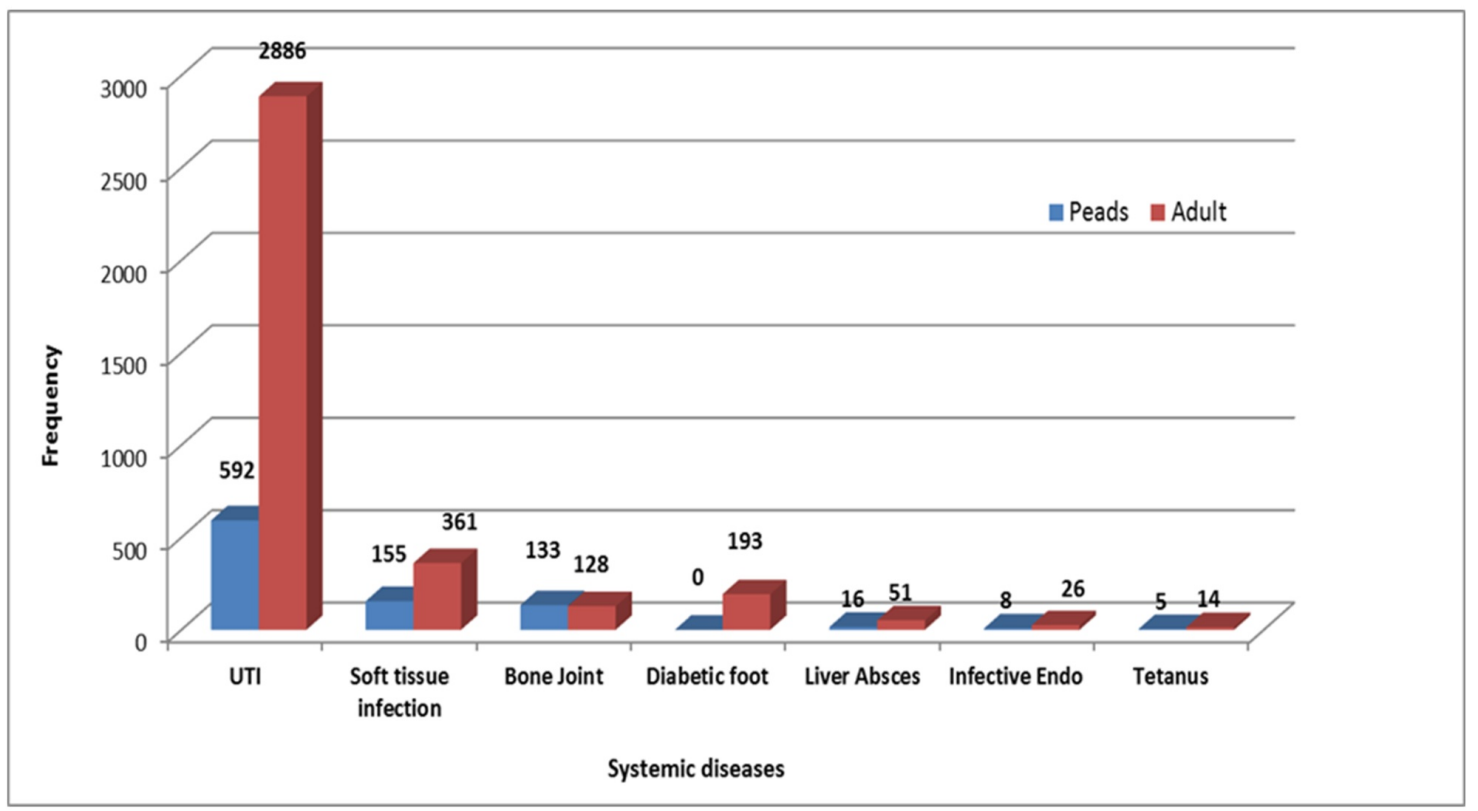
Systemic Diseases Paeds - Paediatrics UTI - Urinary Tract Infection

Among the systemic diseases seen at TIH, urinary tract infections (UTIs) had a disproportionately high incidence, followed by skin and soft tissue infections and while bone and joint infections and diabetic foot had equal incidence. Interestingly, there were still cases of tetanus presented at the hospital (Figure 4).

**Figure 5:**
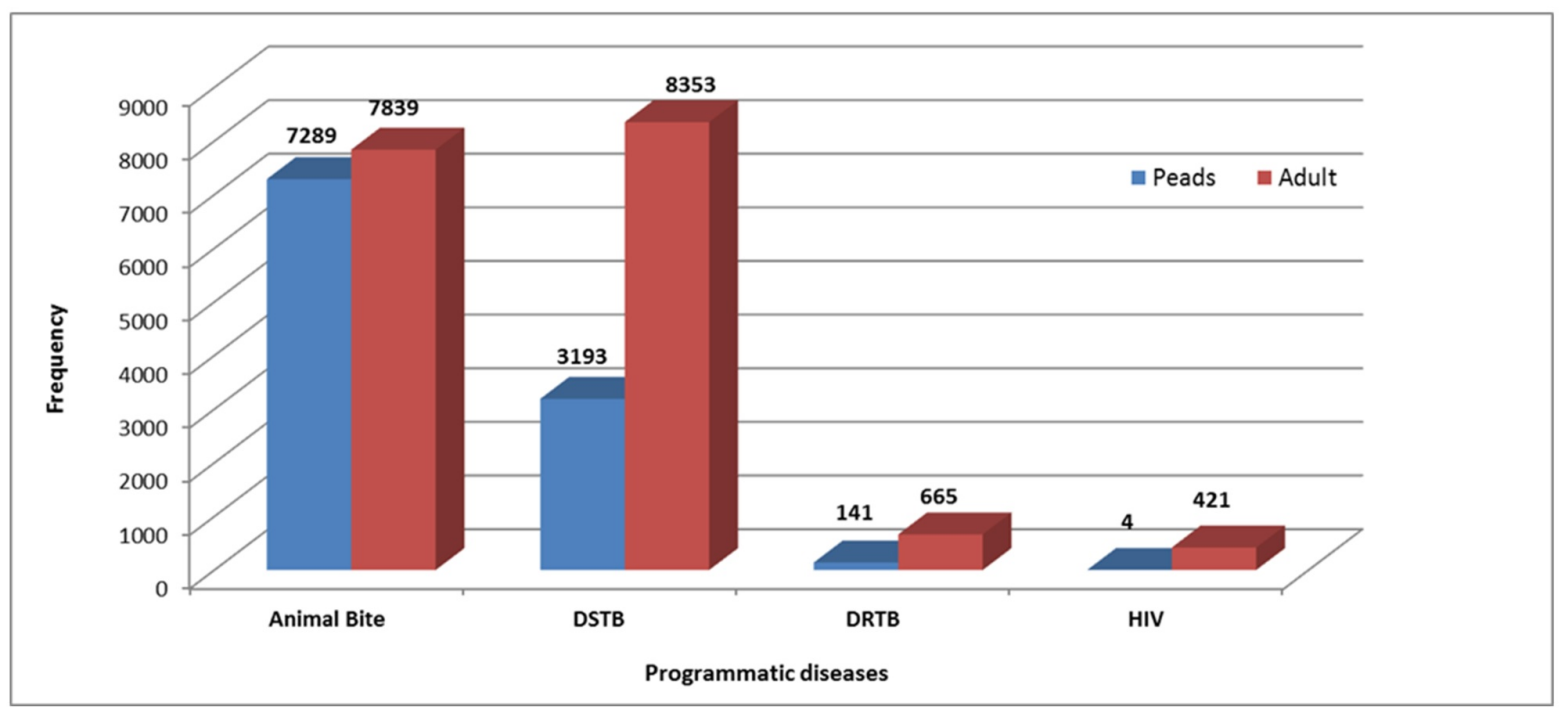
Programmatic Diseases DSTB - Drug-Sensitive Tuberculosis DRTB - Drug-Resistant Tuberculosis HIV - Human Immunodeficiency Virus Paeds - Paediatrics

The rabies prevention (animal bites), TB, and HIV clinics at TIH receive referrals from all over the city and even from remote parts of the country, and hence, their volumes may appear to be unduly high (Figure 5).

In particular, the rabies prevention center (RPC) has one of the largest throughputs in the country; here, WHO recommendations for post-exposure prophylaxis are strictly followed. TIH RPC is also a designated training center for other hospitals and clinics in Sindh province. As evidenced in Table 2, the number of animal bites is very high and are mostly dog bites. No cases of rabies resulted from patients who were provided care at TIH. A total of 55 cases of rabies were referred to the emergency department at TIH from other hospitals and cities where post-exposure prophylaxis was not given or was incomplete over this period.

## Discussion

The model of IDs at TIH may be considered a microcosm of IDs in the rest of the country, and ominously exposes the poor state of healthcare, compounded by overpopulation, overcrowding, poverty, unemployment, water scarcity, drug addiction, and poor governance. Pakistan is fifth in the global burden of tuberculosis [8], and this reflects in our audit with TB as well as viral respiratory tract infections that occur frequently in overcrowded homes, schools, places of work, and among malnourished children and adults. TB is more common among females of reproductive age as compared to males [9], probably because of poorer nutrition, anemia, stigma from the disease, and less likelihood for females to receive prompt medical care.

The high frequency of diarrheal diseases is a result of contaminated water supply and poor personal and environmental hygiene. Diarrheal diseases and typhoid are generally higher in children than in adults, as is indicated in our audit.

Systemic bacterial infections are those affecting individual organs or systems, and are usually acquired in the community, but occasionally in the hospital. Nosocomial infections are becoming increasingly multidrug resistant and lead to high morbidity and even mortality. Among systemic infections, both upper and lower UTIs are very high. Upper UTIs are frequently complicated and resistant to conventional antibiotics [10]. Syed B et al. [10] found that the commonest causes were obstruction of the urinary tract, prior antibiotics use, diabetes mellitus, chronic renal insufficiency, and bladder instrumentation. Women often complained of water scarcity that results in poor personal hygiene. Diabetes mellitus is highly prevalent in Pakistan, its prevalence having increased from 7.6% (5.2 million populace) to 11% in 2011, and is estimated to reach 15% (14 million) by 2030. [11]. It is a frequent cause of UTI, skin and soft tissue and diabetic foot infections that oftentimes lead to limb amputation. The incidence of HIV/AIDS in Pakistan has evolved into a concentrated epidemic through intravenous drug addicts who reuse contaminated syringes. Most such drug users are also co-infected with HCV, syphilis, and TB. Data published between 2010 and 2015 showed that HCV sero-prevalence among the general adult Pakistani population is 6.8%, while active HCV infections were found in approximately 6% of the population [12]. Vector-borne diseases like malaria and dengue are now commonplace and year round; although both are transmitted by the female Anopheles mosquito, their seasonality is slightly different because of different Plasmodium and Dengue virus replication cycles. Chikungunya is not represented in our audit as the outbreak started in Karachi some months after the collection of our data.

TIH is uniquely placed to serve the underprivileged community with quality care at no charge. The outpatient and inpatient numbers in all specialties continue to rise exponentially. The burden of IDs is large and, overall, it reflects the state throughout Pakistan. Poor infrastructure in terms of health, education, and social welfare is the root cause of ill-health and diseases. Failure to provide safe drinking water is responsible for diarrheal diseases, and irresponsible sewage and solid waste disposal results in vector-borne infections. Unchecked population growth with the resultant overcrowding propagates airborne infections as well. The control of infectious threats requires effective surveillance and an analysis of all contributing factors as well as having the means to respond [13].

TB, HIV, and malaria programs in Pakistan function under the global fund and are notifiable by mandate from donor agencies. Except for polio and dengue, there is no active surveillance of the vast array of neglected tropical diseases (NTD) [14]. According to WHO, 64% of IDs are transmitted through vectors. The Centre for Disease Control (CDC) advocates the 'one health' approach through vector control [15-16]. Dog bite-related deaths from rabies are one such NTD that have received scant attention despite its endemicity in urban and rural areas throughout Pakistan [17-19].

Hospitals from across US, Europe, Turkey, Australia, New Zealand, and Singapore - all countries with high standard of living - report either systemic infections such as upper or lower respiratory tract, skin and soft tissue, and sexually transmitted infections among out-patients, and nosocomial infections with drug resistance microorganisms among in-patients [20-25]. There are no reports of tropical infections such as are seen in the subcontinent.

This study serves to bring into focus many prevalent IDs in Pakistan that the lay press has reported on, but no systematic surveillance or studies have been carried out or updated, nor have any interventions been carried out for prevention. Although TB, malaria, and HIV continue to receive much attention by virtue of these infections receiving global attention, no serious attempt has been made at the institutional or governmental levels for widening the scale of EPI programs, safe water provision, or sewage and solid waste management, which could prevent most IDs that afflict the population.

## Conclusions

There is a great need to have an efficient surveillance mechanism of preventable diseases at a national level. Our study highlights the diversity of cases that should direct medical curriculum development, post-graduate training, and health services improvements.
